# Appropriate use of bioresorbable vascular scaffolds in percutaneous coronary interventions: a recommendation from experienced users: A position statement on the use of bioresorbable vascular scaffolds in the Netherlands

**DOI:** 10.1007/s12471-015-0651-3

**Published:** 2015-01-28

**Authors:** Bert Everaert, Cordula Felix, Jacques Koolen, Peter den Heijer, Jose Henriques, Joanna Wykrzykowska, Rene van der Schaaf, Bart de Smet, Sjoerd Hofma, Roberto Diletti, Nicolas Van Mieghem, Evelyn Regar, Peter Smits, Robert-Jan M. van Geuns

**Affiliations:** 1 Thoraxcenter, Erasmus Medical Centre, ’s-Gravendijkwal 230, 3015 GE Rotterdam, The Netherlands; 2 Catharina Hospital, Eindhoven, The Netherlands; 3 Amphia Hospital, Breda, The Netherlands; 4 Academic Medical Centre, Amsterdam, The Netherlands; 5 Onze Lieve Vrouwe Gasthuis, Amsterdam, The Netherlands; 6 Meander Medical Centre, Amersfoort, The Netherlands; 7 Medical Centre Leeuwarden, Leeuwarden, The Netherlands; 8 Maasstad Hospital, Rotterdam, The Netherlands

**Keywords:** Bioresorbable, ABSORB BVS, Consensus, Recommendations, PCI, Coronary artery disease

## Abstract

Percutaneous coronary interventions (PCI) have become a reliable revascularisation option to treat ischaemic coronary artery disease. Drug-eluting stents (DES) are widely used as first choice devices in many procedures due to their established good medium to long term outcomes. These permanent implants, however, do not have any residual function after vascular healing following the PCI. Beyond this initial healing period, metallic stents may induce new problems, resulting in an average rate of 2 % reinterventions per year. To eliminate this potential late limitation of permanent metallic DES, bioresorbable coronary stents or ‘vascular scaffolds’ (BVS) have been developed. In a parallel publication in this journal, an overview of the current clinical performance of these scaffolds is presented. As these scaffolds are currently CE marked and commercially available in many countries and as clinical evidence is still limited, recommendations for their general usage are needed to allow successful clinical introduction.

## Introduction

Percutaneous coronary interventions (PCI) have become a reliable revascularisation option to treat ischaemic coronary artery disease (CAD) [1]. Drug-eluting stents (DES) are widely used as first choice devices in many procedures due to their established good medium to long term outcomes [2]. These permanent implants, however, do not have any residual function after vascular healing following the PCI. Beyond this initial healing period, metallic stents may induce new problems, resulting in an average rate of 2 % reinterventions per year [3]. To eliminate this potential late limitation of permanent metallic DES, bioresorbable coronary stents or ‘vascular scaffolds’ (BVS) have been developed. In a parallel publication in this journal an overview of the current clinical performance of these scaffolds is presented [4]. As these scaffolds are currently CE marked and commercially available in many countries and as clinical evidence is still limited, recommendations for their general usage are needed to allow successful clinical introduction.

## Introduction of new technologies

Continuous technological innovation has contributed to the impressive improvement of medical care over the past decades. On the other hand, many new technologies failed to deliver on their promises and disappeared soon after introduction, such as coronary laser angioplasty and brachytherapy. Moreover, recently, several examples exist of new technologies that were introduced without appropriate recommendations and assessment. A recent example is the introduction of a metal-on-metal hip prosthesis, considered beneficial for younger patients, in which an unexpectedly high rate of device failure was present. Based on similar examples, the Dutch Society of Cardiology (NVVC) adopted policy documents for the introduction of new technologies [5]. Later, the Dutch Order of Medical Specialists in collaboration with ‘Zorginstituut Nederland’ composed a similar document for all medical specialists in the Netherlands [6]. Both documents provide important information on the introduction process for new devices. In these documents a preparation phase is described including a risk analysis and a multidisciplinary cooperation before advising on device introduction. Furthermore, post-introduction outcome registration and reporting are essential to assess for any unexpected adverse events. Also, due to the substantial increase in healthcare costs when CAD patients have to undergo PCI and taking into account that the number of PCIs has more than a doubled over the past 10 years in the Netherlands [7, 8], the cost-effectiveness of any new PCI technology remains an important issue. Regarding the need of adjunctive imaging and supporting techniques for optimal BVS placement, the cost-effectiveness of treating CAD with BVS has yet to be determined in an all-comer patient population.

## Lesion selection

Numerous reviews on the current status of bioresorbable vascular scaffolds (BVS) for PCI have been published. In this journal, we have updated these reviews with the latest data presented during the EuroPCR meeting in Paris from 20 to 23 May 2014. In short, the safety of BVS has once again been confirmed in a large group of patients for non-complex lesions for up to 2 years after scaffold implantation after initial good results for 5 years in smaller groups [9, 10]. Based on these results we think that the use of the Absorb BVS can be considered to be ‘appropriate’ for lesions that were included in the initial ABSORB Cohort A and B trial and the ABSORB Extend registry. The details of these ‘Absorb Extend-like lesions’ are summarised in Table 1. The first randomised study (ABSORB II trial) on whether the Absorb BVS offers an advantage over DES is currently ongoing. The results of the physiological study will be available in 2016. An interim analysis at 1 year of follow-up showed similar rates in a composite clinical endpoint based on death, myocardial infarction and coronary revascularisation [11].

**Table 1:** BVS Extend-like lesions

Absorb extend-like lesions	Exclusion
‘de novo’ lesions	Left main
Diameter 2.3–3.8 mm	Arterial or venous grafts
Length max. 28 mm	In-stent restenosis
One BVS scaffold overlap	Chronic total occlusion
Maximum 2 lesions	Ostial lesions
Stable, unstable or silent ischaemia	Bifurcation lesions with side branches ≥ 2 mm diameter
	Excessive calcification
	High tortuosity
	Visible thrombus
	(N)STEMI
	LVEF < 30 %

*LVEF* left ventricular ejection fraction, *(N)STEMI* (non-)ST-segment elevation myocardial infarction

For patients with more complex lesions, who were excluded from the initial BVS studies, some short-term data were reported during the last EuroPCR meeting. In addition, several medium-sized trials (100–300 patients) with outcome data for up to 12 months were also presented. Based on these data and the experience of the authors, the previous exclusion criteria for Absorb BVS use currently seem outdated. However, as the follow-up period for these more complex lesions as well as patient numbers are still limited, the level of recommendation made cannot exceed ‘probably appropriate’. With this limited evidence in mind, it is important for every operator and PCI centre to keep a registry of patients treated with BVS, including data on patient outcomes, as described in the NVVC guideline on the introduction of new technologies.

Within these real-world registries the number of patients with true complex lesions, such as two scaffold bifurcations, heavily calcified lesions with rotablator lesion preparation and chronic total occlusions, is limited. For these complex lesions no recommendations can be given as the number of patients is too small and the outcomes are still uncertain. Probably, the AIDA (Amsterdam Investigator-initiateD Absorb strategy All-comers) trial, a Dutch multicentre trial with over 2700 patients included, will provide more insights into the use of BVS in these more complex lesions.

Furthermore, two special subsets of lesions should be mentioned: arterial or venous grafts and in-stent restenosis. For both, the current Absorb BVS label (de novo lesions in native vessels) does not apply and at this moment the recommendation for these types of lesions has to be off-label, which should only be deviated from with a clear motivation.

A final—technical—limitation is the overexpansion capabilities of the Absorb BVS that is currently restricted to 0.5 mm. As the largest commercially available Absorb BVS is 3.5 mm at nominal pressure, vessels with a diameter above 4.0 mm (quantitatively measured by quantitative coronary arteriography, intravascular ultrasound (IVUS) and optical coherence tomography (OCT)) should not be targeted because of the greater risk of extensive malapposition (Table 2).

**Table 2:** Lesion selection

Appropriate	Absorb A/B and Extend-like lesions: ‘de novo’ lesions, max. length 28 mm, one stent overlap, max. 2 lesions
Probably appropriate, early evidence	ACS patients, long lesions (> 28 mm), calcified lesions with proper lesion preparation (diameter stenosis < 40 % after preparation), provisional bifurcation treatment (including fenestration into side branch)
Uncertain	Bifurcations requiring a two scaffold approach
	Chronic total occlusion with subintimal crossing
	Extensively calcified lesions where aggressive lesion preparation is necessary
Off-label	In-stent restenosis
	Arterial and venous grafts
	Vessels > 4.0 mm in diameter

*ACS* acute coronary syndrome

## Patient selection

Every introduction of a new technology targets a specific subgroup of patients. With the introduction of DES with higher healthcare costs, initially, only patients with a high risk of early restenosis (diabetic patients, long lesions and small vessels) were selected. Solid clinical data and a price cut paved the way for DES use in the majority of patients undergoing PCI in the Netherlands. As bioresorbable therapy aims to improve patient outcomes after the first year of implantation, patient selection has to take into account other arguments. Although many classical predictors of early restenosis also apply for treatment failure beyond one year (j-Cypher Registry) i.e. diabetes mellitus, renal failure, dialysis and long lesions, some factors are less important for long-term target lesion revascularisation (TLR) such as bifurcation lesions with side branch stenting [12]. It is important to make a differentiation between factors mainly affecting late TLR and those also impacting on patient long-term (> 5 year) survival.

It is therefore essential to appropriately select patients in which BVS may yield the highest beneficial effect on long-time clinical outcome. The American National Cardiovascular Data Registry recently provided valuable information for patient selection [13]. Although a relatively complex model was used, some major points have been identified. Patients above > 80 years, patients with severe renal failure or on dialysis or patients who are in cardiogenic shock at the time of the procedure, have a limited life expectancy and therefore the overall potential long-term benefit of BVS therapy is very limited. Other patient-related conditions, such as diabetes mellitus, body mass index > 40, left ventricular ejection fraction < 40 %, cerebrovascular accident, peripheral artery disease and chronic obstructive pulmonary disease, have a negative impact on the patient’s life expectancy and should lower the upper age limit for patient selection. In Table 3 we provide a simplistic model that can be used for patient selection.

**Table 3:** Patient specific criteria

Optimal	Young patients or with good life expectancy (i.e. > 5 years)	Age < 70 years or Age 70–80 with maximum 1 of PAD, COPD, CVA, renal failure, DM, BMI > 40 or LVEF < 40 %
No potential benefit to be expected	Limited life expectancy (i.e. < 1 or 2 years)	Cardiogenic shock, severe heart failure (EF < 30 %), dialysis

*BMI* body mass index, *COPD* chronic obstructive pulmonary disease, *CVA* cerebrovascular accident, *DM* diabetes mellitus, *LVEF* left ventricular ejection fraction, *PAD* peripheral artery disease

## Technical considerations for BVS implantation

Lesion preparation is especially important, as the current Absorb BVS strut thickness is higher (150 µm) than that of conventionally used DES. Also, before inflation, the initial scaffold diameter is quite large (1.4 mm) which is related to the specific scaffold-related folding characteristics of the Absorb BVS. Therefore, highly calcified or tortuous lesions or lesions with a high degree of angulation can be quite challenging for BVS implantation. However, with extensive lesion pre-dilatation using increasing balloon sizes, even highly calcified lesions can be successfully treated with BVS, although special care has to be paid to a good implantation technique. In summary, the five golden ‘P’s for BVS implantation: Prepare the lesion, Properly size the vessel, Pay attention to the expansion limits of the BVS, Post-dilate the scaffold with a properly sized non-compliant balloon and Pay attention to the DAPT compliance of the patient.

To avoid BVS malapposition, also taking into consideration the current limited sizes in scaffold diameter and length, correct scaffold sizing based on reliable assessment of vessel dimensions is a second important issue. Invasive imaging modalities, such as IVUS and OCT, have been proven to be superior to angiography in providing accurate morphometry, including for estimating vessel diameter and lesion length. OCT is particularly suited to visualise the scaffold struts and their interaction with the vessel wall and can greatly improve the quality of BVS implantation [14]. Before implantation, OCT is indicated to predetermine lesion characteristics, such as lesion length and the amount of calcification, to estimate the optimal scaffold length and to identify the optimal proximal and distal landing zones. OCT after scaffold implantation can be invaluable to guide post-dilatation of the scaffold with properly sized non-compliant balloons to perfect strut apposition, taking into account the expansion limit of 0.5 mm for the Absorb BVS, especially in the initial experience of the operator.

### Challenging lesions

Initial studies with non-complex lesions had a very high procedural success rate. In a more real-world setting lesion preparation, especially for more tortuous and calcified lesions, has proven to be necessary to obtain the same success rates. For truly calcified lesions, rotablation smoothens the atherosclerotic segments and is an invaluable technical aid for procedural success. For less calcified lesions, cutting balloons and the Scoreflex balloon have proven to be of value to appropriately prepare the lesion with full expansion of the pre-dilatation balloons and percentage diameter stenosis < 40 % before BVS implantation. In tortuous vessels the GuideLiner^TM^ or Guidezilla^TM^ guide extension catheters are valuable to increase back-up support and device deliverability. One should keep in mind that these ‘aid devices’ have smaller inner lumens. The 5-in-6F guide extension catheter supports the Absorb BVS 2.5 and 3.0 mm only after preloading. For the Absorb BVS 3.5 mm a 6-in-7F guide extension catheter is necessary.

### Bifurcation lesions

Bifurcation lesions in appropriately selected patients are potentially good candidates for BVS treatment. In these, provisional stenting is the preferred strategy. If side branch treatment is necessary, the proximal optimisation technique (POT) and side branch fenestration with a 2.0 or 2.5 mm balloon at low pressures (max. 8 atms) and final POT is advocated. If balloon fenestration of the side branch is insufficient, eventual bail-out post-dilatation with an undersized balloon and/or scaffolding of the side branch with another BVS or DES and final POT could be needed. Fenestration of side branches with 2.0 and 2.5 mm non-compliant balloons has been tested *in vitro* without strut fractures and is considered safe by many operators. As with metallic stents, scaffold deformation with local malapposition does happen for which post-dilatation is important. As overexpansion capabilities of the Absorb BVS are limited, classical, simultaneous kissing balloon post-dilatation is not recommended. Final invasive imaging optimisation is encouraged. Using BVS, we do not advocate techniques such as the culotte or crush techniques as these could result in ≥ 3 layers of stent struts (≥ 450 µm) with possible compromise of the lumen of the main branch and a high chance of delayed healing of intraluminal uncovered scaffold struts. At this moment the data on BVS bifurcation techniques are still limited compared with metal alloy stent bifurcation techniques.

## Antiplatelet therapy post-PCI

Current guidelines for antiplatelet therapy post-PCI with DES advise dual antiplatelet therapy (DAPT) for 6–12 months for stable angina. For acute coronary syndrome patients, based on the European Society of Cardiology (ESC) non-STEMI and STEMI guidelines, a minimum of 12 months DAPT is advised, preferably with prasugrel or ticagrelor [15, 16]. Some new publications suggest that for second-generation DES, DAPT duration might be shortened [17]. However, most of these studies are retrospective analyses and only a limited number of patients have been analysed for shorter DAPT treatment. For the ABSORB BVS a minimum of 6 months DAPT was stated per protocol, and the majority of patients were on DAPT for 12 months. Based on the design where strut thickness of the ABSORB BVS is similar to first-generation DES, and regarding initial reports on the occurrence of early as well as late stent thrombosis, the best advice for the moment is to prescribe DAPT for 12 months for all patients with ABSORB BVS and to avoid implantation of the ABSORB BVS in patients with a strict indication for oral anticoagulation, as there are currently no data on shorter DAPT in patients on anticoagulants.

## Conclusions

At the start of this new era in interventional cardiology, treating physicians should realise their responsibility for a careful introduction of the technology. This includes the preparation phase and a close follow-up phase for which both the NVVC and the Dutch Order of Medical Specialists have valuable guidelines.

Based on currently reported data and the experience of the authors with the Absorb BVS, some suggestions for the selection of patients and lesion characteristics for correct clinical indications as well as some useful implantation tips and tricks have been made.

### Conflict of interest

Joanna Wykrzykowska and Jacques Koolen received speakers fees from Abbott Vascular. Jose Henriques received an unrestricted educational grant from Abbott Vascular. Peter Smits and Robert-Jan van Geuns received research grants and speakers fees from Abbott Vascular. All the other authors have no conflicts of interest to declare.

## References

[1] Deb S, Wijeysundera HC, Ko DT (2013). Coronary artery bypass graft surgery vs percutaneous interventions in coronary revascularization: a systematic review. JAMA.

[2] Stone GW, Moses JW, Ellis SG (2007). Safety and efficacy of sirolimus- and paclitaxel-eluting coronary stents. N Engl J Med.

[3] Simsek C, Magro M, Boersma E (2010). The unrestricted use of sirolimus- and paclitaxel-eluting stents results in better clinical outcomes during 6-year follow-up than bare-metal stents: an analysis of the RESEARCH (Rapamycin-Eluting Stent Evaluated At Rotterdam Cardiology Hospital) and T-SEARCH (Taxus-Stent Evaluated At Rotterdam Cardiology Hospital) registries. JACC Cardiovasc Interv.

[4] Felix C, Everaert B, Diletti R, et al. Current status of clinically available bioresorbable scaffolds in percutaneous coronary interventions. Neth Heart J. 2015. doi:10.1007/s12471-015-0652-2. 10.1007/s12471-015-0652-2 PMC4352158 25626697

[5] NVVC. Introductie nieuwe technieken in de klinische praktijk. NVVC/NVT. 2011.https://www.nvvc.nl/media/files/03_Introductie_nieuwe_interventietechnieken_in_de_klinische_praktijk.pdf.

[6] Zorginstitituut Nederland. http://www.zorginstituutnederland.nl/kwaliteit/projecten/leidraad+innovaties.

[7] Leening MJ, Siregar S, Vaartjes I (2014). Heart disease in the Netherlands: a quantitative update. Neth Heart J.

[8] Soekhlal RR, Burgers LT, Redekop WK, Tan SS (2013). Treatment costs of acute myocardial infarction in the Netherlands. Neth Heart J.

[9] Onuma Y, Dudek D, Thuesen L (2013). Five-year clinical and functional multislice computed tomography angiographic results after coronary implantation of the fully resorbable polymeric everolimus-eluting scaffold in patients with de novo coronary artery disease: the ABSORB cohort A trial. JACC Cardiovasc Interv.

[10] Serruys PW, Onuma Y, Garcia-Garcia HM (2014). Dynamics of vessel wall changes following the implantation of the absorb everolimus-eluting bioresorbable vascular scaffold: a multi-imaging modality study at 6, 12, 24 and 36 months. EuroIntervention.

[11] Serruys PW, Chevalier B, Dudek D, et al. A bioresorbable everolimus-eluting scaffold versus a metallic everolimus-eluting stent for ischaemic heart disease caused by de-novo native coronary artery lesions (ABSORB II): an interim 1-year analysis of clinical and procedural secondary outcomes from a randomised controlled trial. Lancet. 2014. doi:10.1016/S0140-6736(14)61455-0. 10.1016/S0140-6736(14)61455-0 25230593

[12] Kimura T, Morimoto T, Nakagawa Y (2012). Very late stent thrombosis and late target lesion revascularization after sirolimus-eluting stent implantation: five-year outcome of the j-Cypher Registry. Circulation.

[13] Wu C, Camacho FT, King SB (2014). Risk stratification for long-term mortality after percutaneous coronary intervention. Circ Cardiovasc Interv.

[14] Allahwala UK, Cockburn JA, Shaw E, et al. Clinical utility of optical coherence tomography (OCT) in the optimisation of Absorb bioresorbable vascular scaffold deployment during percutaneous coronary intervention. EuroIntervention. 2014. [Epub ahead of print]. 10.4244/EIJV10I10A190 24647105

[15] Wiviott SD, Braunwald E, McCabe CH (2007). Prasugrel versus clopidogrel in patients with acute coronary syndromes. N Engl J Med.

[16] Wallentin L, Becker RC, Budaj A (2009). Ticagrelor versus clopidogrel in patients with acute coronary syndromes. N Engl J Med.

[17] Colombo A, Chieffo A, Frasheri A (2014). Second generation drug eluting stent implantation followed by six versus twelve month dual antiplatelet therapy: the SECURITY randomized clinical trial. J Am Coll Cardiol.

